# Estimating Household and Community Transmission of Ocular *Chlamydia trachomatis*

**DOI:** 10.1371/journal.pntd.0000401

**Published:** 2009-03-31

**Authors:** Isobel M. Blake, Matthew J. Burton, Robin L. Bailey, Anthony W. Solomon, Sheila West, Beatriz Muñoz, Martin J. Holland, David C. W. Mabey, Manoj Gambhir, María-Gloria Basáñez, Nicholas C. Grassly

**Affiliations:** 1 Department of Infectious Disease Epidemiology, Imperial College London, London, United Kingdom; 2 London School of Hygiene and Tropical Medicine, London, United Kingdom; 3 Dana Center for Preventive Ophthalmology, Johns Hopkins University, Baltimore, Maryland, United States of America; University of California San Francisco, United States of America

## Abstract

**Introduction:**

Community-wide administration of antibiotics is one arm of a four-pronged strategy in the global initiative to eliminate blindness due to trachoma. The potential impact of more efficient, targeted treatment of infected households depends on the relative contribution of community and household transmission of infection, which have not previously been estimated.

**Methods:**

A mathematical model of the household transmission of ocular *Chlamydia trachomatis* was fit to detailed demographic and prevalence data from four endemic populations in The Gambia and Tanzania. Maximum likelihood estimates of the household and community transmission coefficients were obtained.

**Results:**

The estimated household transmission coefficient exceeded both the community transmission coefficient and the rate of clearance of infection by individuals in three of the four populations, allowing persistent transmission of infection within households. In all populations, individuals in larger households contributed more to the incidence of infection than those in smaller households.

**Discussion:**

Transmission of ocular *C. trachomatis* infection within households is typically very efficient. Failure to treat all infected members of a household during mass administration of antibiotics is likely to result in rapid re-infection of that household, followed by more gradual spread across the community. The feasibility and effectiveness of household targeted strategies should be explored.

## Author Summary

Trachoma is a major cause of blindness worldwide and results from ocular infection with the bacterium *Chlamydia trachomatis*. Mass distribution of antibiotics in communities is part of the strategy to eliminate blindness due to trachoma. Targeted treatment of infected households could be more efficient, but the success of such a strategy will depend on the extent of transmission of infection between members of the same household and between members of the community. In this work, we estimated the magnitude of household and community transmission in four populations, two from The Gambia and two from Tanzania. We found that, in general, transmission of the bacteria within households is very efficient. In three of the four populations, persistent infection within households was predicted by the high level of household transmission (a phenomenon observed in longitudinal studies of trachoma). In all of the studied populations, individuals who live in households with more individuals contribute more to the number of new infections in the community than those who live with fewer individuals. Further studies are required to identify and examine household-targeted approaches to treatment.

## Introduction

Trachoma is the leading cause of infectious blindness worldwide. Eight million people are visually impaired from the disease and a further 46 million people with active disease are in need of treatment to prevent blindness [1]. Mass drug administration (MDA) with antibiotics (predominantly azithromycin but also topical tetracycline) is one of the four arms of the SAFE strategy, advocated by the World Health Organization (WHO) to control trachoma with the aim of Global Elimination of Blinding Trachoma by 2020 (GET 2020). Large scale vertical control programmes currently operate, such as those through the partners of the International Trachoma Initiative, and control efforts are expected to expand when trachoma control is integrated with that of other neglected tropical diseases [2].

The presence of active disease is currently used to guide trachoma control programs and to evaluate the success of interventions. The WHO advises that if the prevalence in a district of trachomatous inflammation follicular (TF) in a district among 1–9 year-old children is ≥10%, annual treatment of the district along with face-washing and environmental improvement should occur for at least three years until the prevalence of active disease in that age group is reduced to less than 5% [3]. However there is a loose relationship between an individual showing signs of active disease and being infected with the causative bacterium of trachoma, *Chlamydia trachomatis*. There is typically a lag before the appearance of active disease after an individual has been infected and a persistence of active disease after infection resolves [4],[5]. Signs of conjunctival inflammation may also be the result of other bacterial infections or mechanical irritation [6] and even after infection is eliminated from a community, some individuals may still show signs of active disease [7]. Therefore the proportion of individuals with active disease may not correspond to the proportion of individuals with infection. This was recently illustrated by a study in The Gambia in which the overall prevalence of infection among children under 10 years of age in two regions was 0.3% based on qualitative PCR testing of conjunctival swabs, whereas the prevalence of active disease in this age group was 10.4% [8].

Control programmes that have used MDA as part of their control strategy have had some success [9], and people may also benefit from other bacterial infections being cleared by the antibiotic. Although most antibiotics are currently donated, donation is not universal and is likely to be time-limited. There are also many costs associated with delivering antibiotics in rural settings [10],[11]. Furthermore, MDA results in many uninfected individuals receiving treatment and could promote antibiotic resistance among other bacterial infections such as *Streptococcus pneumoniae* [12]. Targeted treatment to those infected would reduce the number of drug doses required, potentially reducing the cost of MDA. However, the loose relationship between infection and active disease makes targeted treatment of individuals with active disease ineffective at the population level. Targeting households with at least one member with active disease may be more effective since infection clusters by household [13] and so asymptomatic infections are more likely to be treated. In The Gambia, this strategy has been used as national policy in communities with less than 5% of TF among children aged 1–9 years old (Personal communication, Mr Ansumana Sillah, Manager, Gambian National Eye Care Programme).

Clustering of active trachoma disease by household has been shown to occur in a number of communities [13]–[17] and individuals living with people who have active trachoma are more likely to have active disease than individuals who live with individuals without active disease [15], [18]–[20]. Furthermore, in Jali village in The Gambia, the same serovar of *C. trachomatis* was predominantly found within a household even though three serovars were present in the community [21], suggesting that transmission between members of the same household is more common than between other members of the community with different serovars. However, the rates of transmission between individuals of the same household and between members of the same community have not been estimated and little is known about the likely impact of targeted treatment of households on transmission of *C. trachomatis*.

Here we examine the contribution of transmission between members of the same household and that between households of the same population to the incidence of ocular *C. trachomatis* infection using cross-sectional data on the prevalence of infection from four endemic communities, two in West Africa (The Gambia) and two in East Africa (Tanzania). We discuss the implications of our findings for the resurgence of infection after community-wide treatment and the potential for targeted treatment of households to reduce infection efficiently.

## Methods

### Data

Individuals of all ages from four endemic populations (Upper Saloum District and Jali village in The Gambia and Kahe Mpya and Maindi villages in Tanzania) were examined and conjunctival swabs taken to test for the presence of chlamydial infection using PCR amplification of a target sequence in the common cryptic plasmid of the bacteria. In one community, Maindi village, the presence of infection was based on quantitative PCR amplification of the *omp*1 gene. Detailed information on the bedroom (Upper Saloum District, Kahe Mpya sub-village and Jali village only), household (Upper Saloum District, Kahe Mpya sub-village and Maindi village only), compound (Jali village and Upper Saloum district only), balozi (Kahe Mpya sub-village and Maindi village only) and village (Upper Saloum district) of the individuals examined was recorded; along with a number of other risk factor for trachoma and clinical signs of disease. Characteristics of these populations and detailed methods have been reported previously [15],[19],[22],[23].

The study in Upper Saloum district was approved by the Gambian Government/Medical Research Council Joint Ethics Committee (SCC 856) and the London School of Hygiene and Tropical Medicine Ethics Committee. Written informed consent was obtained from all individuals. The Kahe Mpya study was approved by the London School of Hygiene and Tropical Medicine committee and the Kilimanjaro Christian Medical Centre, Tanzania. Written consent was obtained. The study in Maindi village was approved by the Johns Hopkins Institute Review Board and the Tanzanian National Institute for Medical Research; all participants provided oral informed consent. Both IRBs approved oral informed consent because many of the rural villagers are illiterate and asking them to sign a document they cannot read is unethical; in the past, unscrupulous persons have had them sign official “documents” that were really signing away their land. Oral consent was witnessed and documented by a member of the team on a study document. These three studies were done in accordance with the Helsinki Declaration. The study in Jali received ethical approval from the joint Gambia Government and Medical Research Council Ethics Committee (SCC 508). All subjects gave oral informed consent that was witnessed and signed by the witness following the standard consent procedures at the time.

### Household Model of Transmission

Trachoma is a disease in which a fully protective immune response against re-infection is not elicited and so individuals can be repeatedly infected [18],[24]. We therefore chose to describe transmission using a simple Susceptible→Infected→Susceptible (SIS) model, in which the population is categorised into two groups - individuals susceptible to infection (S) or infected individuals (I) - and infected individuals recover to become susceptible again. Household SIS models have been previously examined by Ball [25] and Neal [26].

The probability that a household of size 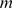 has 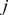 infected individuals (and 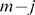 susceptible individuals) at time 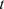 is given by 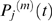. A susceptible individual can be infected from either an infected member of the community (global transmission) at a rate: 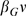, in which 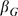 is the global transmission coefficient and 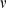 is the prevalence of infection in the community; or from an infected member of the same household (local transmission) at a rate: 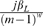, in which 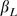 is the local transmission coefficient. 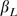 is multiplied by either the number of infected individuals in the household, 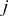, if transmission is assumed to be density dependent (the average number of contacts per individual increases with household size, corresponding to 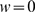), or the fraction of infected individuals in the household 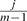, representing that the average number of contacts per individual is constant, regardless of household size, and corresponding to 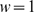. The parameter 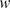 is therefore the coefficient for density dependence, which in the application described we allow to vary on a continuous scale with 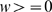.

Individuals recover from infection at a rate 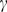, taken as the reciprocal of the average duration of infection. Births and deaths are not included in the model because the duration of infection is relatively short compared to the average human life expectancy.

We can write the difference-differential equation for 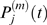,
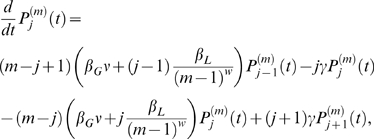
(1)where 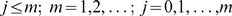 and 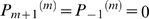.

At endemic equilibrium, assuming the number of households 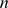 is large (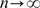), solving 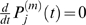, leads to the recursion:
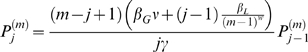
(2)

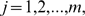
where
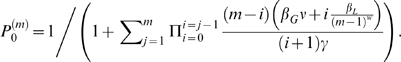
(3)


The prevalence of infection in the community described by equations (2) and (3) is
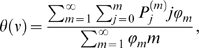
(4)where 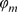 is the fraction of households of size 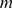 in the population. Solving equations (2) and (3) therefore requires the implicit equation 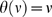 to be satisfied at equilibrium.

An epidemic can occur when the household basic reproduction number 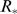 is greater than 1 [25]. 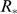 is defined as the mean number of households infected following the introduction of a single infected individual to a randomly chosen household. It is analogous to the basic reproduction number 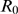 in a non-structured, randomly mixing population [27]. If a household of size 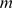 is initially infected then 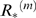 is
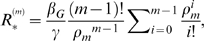
(5)where
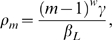
(6)and 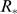 is the average across all individuals according to their probability of being in a household of a given size,
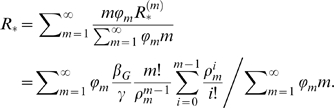
(7)


### Estimation

Maximum likelihood was used to estimate 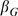, 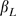 and 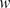 simultaneously. The likelihood, 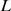, of a household of size 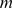, with 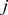 individuals infected is given by 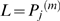 and the total log-likelihood is the summation of 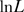 across all households.

The duration of infection was assumed to be 17.2 weeks based on cohort studies of infection with frequent follow-up [4] and 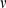 was taken to be the prevalence of infection in the cross-sectional survey (*i.e.* infection in the communities, prior to antibiotic intervention, is assumed to be at endemic equilibrium). The sensitivity of the estimates to the assumed duration of infection was examined for a range of plausible values (12–24 weeks) [4]. Confidence intervals (CI) for each parameter were calculated by assuming that 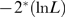 is approximately 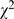 (chi-squared) distributed [28]. We therefore tested the hypothesis of density dependence in the contact rate by estimating parameter 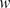 and its confidence intervals; the null hypothesis of density dependence (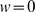) was contrasted with the alternative hypothesis of frequency dependence (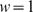), by ascertaining whether the confidence intervals around the estimate included 0 or 1.

A small number of individuals were not tested for the presence of infection, due to refusal or because they were away travelling. The sensitivity of the estimates to the inclusion of these individuals as members of the household such that they may have contributed to transmission was examined (Text S1). If there were 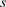 members of a household tested for infection and an additional 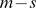 individuals who were not tested for infection but who contribute to transmission, the probability that 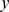 individuals were found positive in the sample, given that 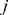 members of the overall household of size 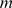 were actually infected (according to a hypergeometric distribution [29]) is:
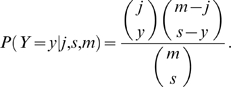
(8)


In this case the likelihood for each household can be modified such that
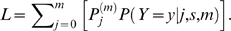
(9)


This assumes that infected individuals are equally likely to be sampled as uninfected individuals. The sensitivity of this assumption was explored using the non-central hypergeometric distribution [29] (Text S1).

The impact of different definitions of a ‘household’ on the estimates of 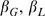 and 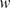 was examined, from bedroom, household, compound and village for the Upper Saloum District; room and compound for Jali village; room, kaya and balozi for Kahe Mpya sub-village and kaya and balozi for Maindi village. (See below in the Results section for the definitions of ‘kaya’ and ‘balozi’).

### Model Fit

The appropriateness of the household SIS model of *C*. *trachomatis* transmission was assessed by simulating the number of people infected at endemic equilibrium and the household to which they belong under the model using the estimated parameters and assuming a negative binomial distribution for the underlying household size distribution (with inverse overdispersion parameter 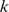 equal to (95% CI denoting 95% confidence intervals): 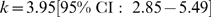, and 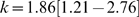, for respectively Upper Saloum district and Jali village (The Gambia), and 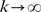 for both Kahe Mpya and Maindi village (Tanzanaia), where 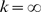 corresponds to a random or Poisson distribution; see Text S1). The probability mass function used for the negative binomial is [30]:
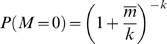
(10)and when 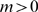


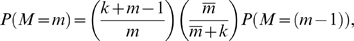
(11)where 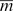 is the (arithmetic) mean household size (Table 1). Comparison of the model simulations with the observed data was based on the mean intraclass correlation coefficient for the prevalence of infection within households (ICC). The ICC provides a quantitative measure of similarity between individuals within groups and is based upon the comparison of within- and between-group sums of squares from an analysis of variance [31]. One thousand stochastic simulations were run for each setting using the numerical integration package Berkeley Madonna [32].

**Table 1:** Demographic and prevalence data from the four populations examined for ocular *Chlamydia trachomatis*.

Population and reference of study	Year at baseline	No. individuals in population	No. individuals tested for chlamydial infection at baseline	Prevalence of infection (%)	Mean household size (number)	Percentage of households infected (%)
14 villages, Upper Saloum District, The Gambia [15]	2001	1595	1319	7.2	13.6	24.8
Jali village, Kiang West District, The Gambia [22]	1991	844	752	22.1	17.3	73.5
Sub-village of Kahe Mpya, Rombo District, Tanzania [23]	2000	978	956	9.5	5.3	30.0
Maindi village, Kongwa District, Tanzania [19]	2000	1017	783	36.0	4.7	60.4

NOTE: The household unit for Jali village was a compound as household data was unavailable.

## Results

### Community Structure and Prevalence of Infection

In The Gambia one household or a cluster of households forms a compound, a unit which is fenced off from the rest of a community. In Upper Saloum district the household unit ranges from 1–55 individuals and the compound ranges from 2–77 individuals. In Jali village the compound unit ranges from 4–70 individuals (household data unavailable). In Tanzania, the household unit is the ‘kaya’, (ranging from 1 to 14 individuals) and on average the unit is smaller than the household unit in The Gambia (Table 1). Kayas which are situated within the same geographical zone are grouped into a ‘balozi’ and share a balozi leader. The number of individuals examined in each community along with the prevalence of infection among households and among individuals is given in Table 1.

### Estimates of Household Transmission

**Figure 1:**
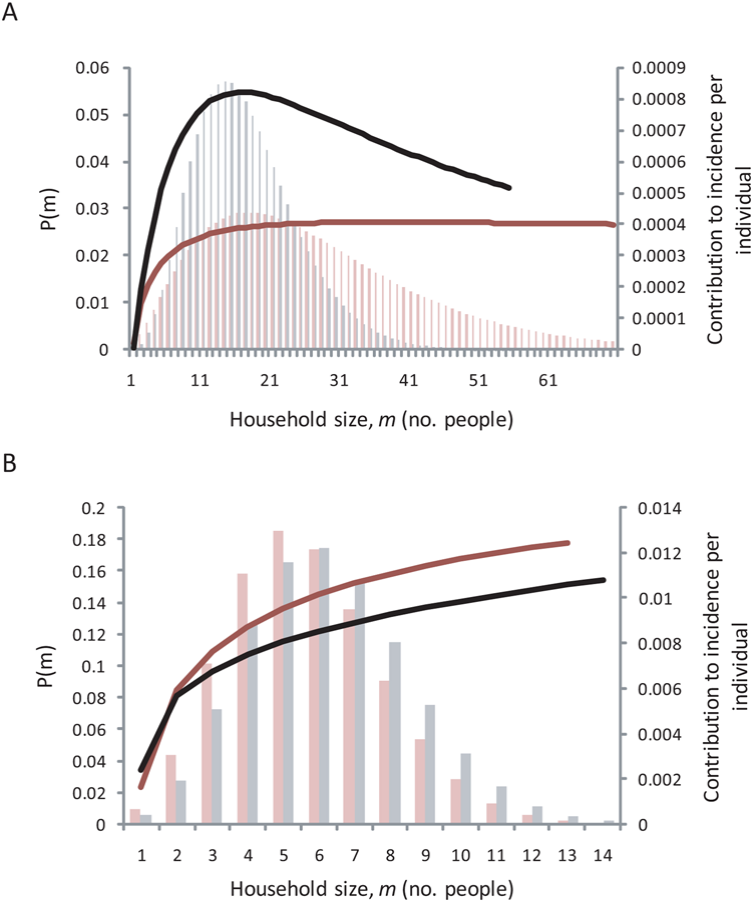
Proportion of incidence contributed per individual from a household of size 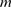 (solid line) and the probability distribution of a randomly chosen individual belonging to a household of that size, P(m), based on the negative binomial distribution in (A) The Gambia – the black lines correspond to Upper Saloum district and the red lines correspond to Jali village; and (B) Tanzania – the black lines correspond to Kahe Mpya sub-village and the red lines correspond to Maindi village.

The estimates for the global and local transmission coefficients (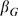 and 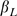) along with the density-dependent coefficient, 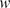 and the household reproduction number 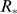 are given in Table 2 along with their 95% confidence interval. In Jali the compound unit was used because household data were unavailable. The estimates of 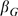 and 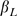 were sensitive to changes in the duration of infection, whereas the estimates of 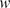, 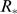 and the ratio 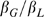 were not affected by changes in the duration of infection (Text S1). Estimates of 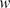 were close to 1, and in all of the four populations the 95% CIs included 1, consistent with frequency-dependent transmission, such that the number of contacts made by an infected individual was not larger in bigger households. Estimates of the rate of household transmission were large and 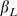 was greater than 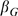 in three of the four populations. In all four, individuals from larger households were estimated to contribute more to incidence than those from smaller households (Figure 1). This effect reverses somewhat at very large household sizes in Upper Saloum District where the estimate of 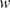 (>1) is consistent with a decline in the number of infectious contacts with increasing household size. An average of 71% of incident infections were the result of transmission within the household (with a range of 48%–91%) in the four populations.

**Table 2:** Maximum likelihood estimates of the transmission parameters in four populations of West and East Africa.

Population	Global transmission coefficient, *β_G_*	Local transmission coefficient, *β_L_*	Coefficient for density dependence, *w*	R_*_
	[95% CI]	[95% CI]	[95% CI]	
14 villages, Upper Saloum District, The Gambia	0.29 [0.16–0.51]	7.09 [3.58–13.73]	1.22 [0.99–1.45]	1.25
Jali village, Kiang West District, The Gambia	0.76 [0.39–1.40]	4.01 [1.81–7.38]	1.05 [0.84–1.23]	2.81
Sub-Village of Kahe Mpya, Rombo District, Tanzania	1.73 [1.18–2.37]	1.57 [0.29–5.31]	0.89 [0.06–1.63]	1.18
Maindi village, Kongwa district, Tanzania	1.70 [1.15–2.46]	3.06 [1.14–6.18]	0.88 [0.41–1.26]	2.65

NOTE: The transmission parameters were obtained by fitting the model of household transmission of *C. trachomatis*, described in the main text, to the baseline data summarised in Table 1.

The estimate of 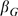 increased as the definition of the household unit became smaller in size (from village to compound; balozi to household; kaya to room) and the estimates of 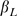 and 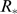 decreased (except for 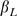 in the Upper Saloum District) and 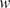 remained approximately constant (Text S1). Exclusion of the individuals who were not examined at the moment of sampling but were members of households in the four populations does not change the parameter estimates significantly (Text S1). Assuming infected individuals to be more or less likely to be sampled did not alter the parameter estimates significantly either (Text S1).

### Model Fit

The average ICCs from the model simulations were in agreement with the ICCs calculated from the data, suggesting that the simple SIS model of household transmission captures much of the dynamics of *C. trachomatis* infection in these communities (Table 3).

**Table 3:** Comparison of the ICC from four populations endemic for trachoma with the mean simulated ICC.

Community	ICC from data	Mean simulated ICC [95% CI]
14 villages, Upper Saloum District, The Gambia	0.26	0.23 [0.23–0.24]
Jali village, Kiang West District, The Gambia	0.10	0.08 [0.08–0.08]
Sub-village of Kahe Mpya, Tanzania	0.11	0.12 [0.12–0.13]
Maindi village, Tanzania	0.14	0.15 [0.150–0.16]

NOTE: ICC = Intraclass correlation coefficient, with the mean ICC calculated from running 1000 stochastic simulations. The stochastic simulations used the estimated household transmission parameters (Table 2) and the fitted household size distribution (main text).

## Discussion

Clustering of infection by household is an important epidemiological feature of many communicable diseases and is thought to be a key characteristic of trachoma. However, the magnitude of transmission of *C. trachomatis* between individuals belonging to the same household and that between individuals living in different households but the same community have not, to our knowledge, previously been estimated. Here they are estimated in four different populations by fitting a household model of transmission to prevalence data using maximum likelihood estimation. In these communities an average of 71% of incident infections were the result of transmission within the household, indicating the important role of household transmission in the repeat infections with *C. trachomatis* that result in progression to trachomatous scarring and blindness. In all four populations, individuals who live in relatively large households (*i.e.* with many individuals) contribute more to incidence than those who live in households with fewer individuals. Further to this, in the two Gambian populations and in Maindi village, Tanzania, the household transmission coefficient was estimated to be greater than the rate of recovery from infection, such that sustained transmission within the household is possible (Table 2) In other words, the expected duration that a household is infected will be significantly longer than an individual's duration of infection, despite eventual stochastic extinction. The resulting persistence of infection within households permits epidemic spread on average following the introduction of infection into a household (*i.e.* 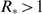) even if the community transmission coefficient is low. For this reason, the dynamics of infection following community-wide treatment may be different from that expected based on a non-structured mathematical model of transmission [33],[34].

The persistence of infection within households due to efficient household transmission and repeated infection of household members has been observed during follow-up of endemic communities [35],[36]. Gradual spread across communities over the course of about one year has been observed following community-wide treatment in several studies [19], [37]–[39]. Such gradual spread is difficult to reconcile with the estimated, rather short duration of infection of individuals with ocular *C. trachomatis* unless the importance of household transmission is considered. In comparison to the other three populations, in Kahe Mpya sub-village, Tanzania, the estimated household transmission coefficient was lower than both the global transmission coefficient and the rate of recovery from infection 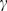, indicating that in this community, persistence within households does not occur. This may be the result of a difference in social behaviour of this community or perhaps a difference in the fly population that may act as a mechanic vector of trachoma. Interestingly, infection was successfully eliminated from this community after two mass treatments with azithromycin and multiple targeted treatment of active disease with topical tetracycline at follow-up time points [7].

The variation of the results within the two studies countries and the small number of populations studied in each country make inter-country comparisons difficult. Generally, the two populations studied in The Gambia were estimated to have higher local (household) and lower global (community) transmission compared to the two populations in Tanzania *i.e.* household transmission was estimated to be more efficient in The Gambia than Tanzania. The higher household transmission in The Gambia is not intuitive from the differences in geographical distances between households in the two countries. Households are further apart in the Tanzanian populations than those in The Gambia and from this one may think community transmission to be lower in Tanzania. However our work indicates community transmission to be higher in Tanzania. This may be explained by differences in their community structure: Individuals in The Gambia live in much larger households which cluster together to form large compounds. The larger size of the living unit may limit the number of contacts made with the rest of the community therefore sustaining transmission within the household. Moreover, the results of the sensitivity analysis of the household unit definition indicate that the smaller the unit, the higher the amount of community transmission required to sustain transmission. The estimates of the transmission coefficients are less certain for the Tanzanian populations than for the Gambian ones because there are fewer large households, which contribute most information to the estimate of household transmission.

Estimates of the density dependence of transmission found that 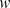 was close to 1 in all communities, with the 95% confidence intervals containing 1 (Table 2). This indicates that individuals typically have a fixed number of contacts per household regardless of household size (*i.e.* the risk of infection is proportional to the fraction of infective individuals in a household, rather than the number). This phenomenon has also been shown for other infections, such as *Streptococcus pneumoniae* and influenza virus [40],[41]. The estimate of 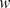 from the data from Upper Saloum district is slightly higher than the other estimates (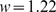), resulting in a slight decline in the number of contacts per individual with household size, although the confidence intervals include 1 (Figure 1).

The household model used in this work assumes that all individuals mix homogeneously outside their household at the same rate (specific to each setting), such that each household is at equal risk of infection. It ignores any protective immunity against re-infection, does not include infection with different serovars, and assumes that an individual's age does not affect their duration of infection or risk of acquiring infection. It also assumes that each infected individual is equally infectious and does not therefore take into account that some individuals harbor a much higher number of bacteria than others. These assumptions are simplifications of disease transmission and natural history, and in particular, neglect the differences between adults and children in their contribution to transmission. Children have a longer duration of infection and a higher prevalence of infection than adults. Children may also have a different within/between household contact pattern than adults. However, the correspondence between the model simulations and the data indicate that the model is a reasonable description of the household transmission of ocular chlamydial infection. Further work will examine in more detail the contribution of individuals of different ages to the transmission of ocular *Chlamydia* within households. We have assumed accurate testing of individuals for ocular chlamydial infection and that there was no contamination of the conjunctival swabs. Although, cross-contamination of samples is a risk when using PCR techniques, standard precautions were taken to prevent this [22],[42].

The strategy of mass antibiotic treatment to control trachoma can be costly [10], may result in antibiotic treatment of uninfected individuals and may increase the chance of antibiotic resistance developing, as observed for other bacterial infections [43]–[46]. A control approach which minimises the number of antibiotic doses given out in a community but still has similar effects in reducing prevalence in a community compared to mass distribution would therefore be advantageous. In this paper we have quantified the amount of household and community transmission for the first time and have shown that this leads to persistently infected households in 3 of the 4 study populations. Furthermore, in all four such populations, individuals living in larger households contributed more to transmission than those living in smaller households (Figure 1). This suggests a potential role for the targeted treatment of households more likely to harbor infection. Two field studies have explored the use of the household as the unit for targeting treatment and come to differing conclusions. In Nepal, the reduction in prevalence of active disease after community-wide treatment and after targeted treatment of households containing children showing active disease were not significantly different [47]. In Mali, treatment of those households where at least one child had active disease was significantly less effective at controlling active disease than mass treatment [48]. However, these two studies used active disease as an indicator for treatment and therefore may have missed some children who would have been infected but without showing signs of active disease [4],[5]. Other methods of targeted treatment could also be explored, such as the use of a dipstick assay for rapid diagnosis of the presence of infection, which is currently being developed [49].

The critical role of the household in the transmission and persistence of trachoma demonstrated by our study, along with the high cost of community-wide antibiotic treatment, highlight both the potential and the need for targeted approaches for the treatment of ocular chlamydial infection. Further studies are needed to identify efficient and effective methods to achieve this.

## Supporting Information

Text S1 (0.18 MB DOC)
