# Supplementary material for: Estimating Household and Community Transmission of Ocular Chlamydia trachomatis
Source: PLoS Negl Trop Dis. 2009 Mar 31;3(3):e401. doi: 10.1371/journal.pntd.0000401 (PMC2655714; doi:10.1371/journal.pntd.0000401)
Supplement: Text S1 — (0.18 MB DOC) [file pntd.0000401.s001.doc]

Supplementary Information

# Fitting the Negative Binomial distribution to the Household Size distribution

The negative binomial distribution [1] was fitted to the household size distribution of each community by obtaining maximum likelihood estimates of the inverse overdispersion parameter, $k$(when $k\to0$the distribution is overdispersed and when $k\to\infty$the distribution follows a Poisson distribution) (Text S1 FIGURE 1). The mean household size was calculated for each community. Confidence intervals for the estimates of $k$ were calculated by assuming that -2^*^($\ln L$) is approximately $\chi$^2^ (chi-squared) distributed.

# Sensitivity Analysis

Definition of Household unit

The impact of different definitions of the ‘household’ on the estimates of$\beta_{G}$, $\beta_{L}$and $w$ was examined, from bedroom, household, compound and village for the Upper Saloum District; room and compound for Jali village; room, kaya and balozi for Kahe Mpya sub-village and kaya and balozi for Maindi village. The transmission parameters were re-estimated using each of the different units for each setting (Text S1 TABLE 1). Large values of $R_{*}$were obtained when using the village as the definition of the household unit in Upper Saloum district and when using the balozi as a definition of a household unit in Maindi village because in these settings the average size of the ‘household’ unit is relatively large (316 individuals in Upper Saloum district and 68 in Maindi village) and $\beta_{L}$ is much greater than the recovery rate. Therefore infection persists in these households and they are so large many secondary households are infected. However the model assumes that the number of units tends to infinity and because there are very few villages (14) or balozis (15) in the data it is not suitable to use the model when considering these units.

Missing Data

Some individuals from the communities were absent at time of examination. The sensitivity of the transmission parameter estimates to the inclusion of these individuals as members of the household such that they may have contributed to transmission was examined by estimating the parameters both by including these individuals as members of the household (TABLE 2 main text), with a probability $P\left(Y=y\mid j,s,m\right)$ that they are infected, and excluding them (Text S1 TABLE 2) (*i.e.* reducing the size of each household).

When accounting for the missing individuals, we assume individuals were missing at random i.e. infected individuals were equally likely to be sampled as uninfected members. The sensitivity of this assumption was examined by re-estimating the parameters if infected individuals were twice of half as likely to be sampled using the non-central (Fisher) hypergeometric distribution (Text S1 TABLE 3) and these estimates were compared to the original estimates (which assume infected and uninfected individuals are equally likely, probability ratio of uninfected: infected individuals being sampled = 1).

Duration of Infection

The sensitivity of the estimates to the assumed duration of infection was examined for a range of plausible values (12 – 24 weeks). The parameters were re-estimated using values within this range. The ratio of $\beta_{G}/\beta_{L}$ was used to show whether the relationship between these two parameters change when using different values of the duration of infection (Text S1 FIGURE 2).

Text S1 FIGURE 1 Fitted and observed household distributions. The inverse overdispersion parameter, *k*, was estimated to be with 95% confidence intervals (CI)$k$ = 3.95 [95% CI: 2.85 – 5.49], $k$= 1.86 [1.21 – 2.76], $k$= $\infty$ and $k$= $\infty$ for, respectively, Upper Saloum district, Jali village, Kahe Mpya and Maindi village, where $k=\infty$ corresponds to a random or Poisson distribution.

| Community | Unit | Fraction of units infected | *β_G_* [95% CI] | *β_L_* [95% CI] | *w* [95% CI] | *R** |
| --- | --- | --- | --- | --- | --- | --- |
| 14 villages, Upper Saloum district,  The Gambia | Village | 0.50 | 0.03 [0.006 – 0.09] | 6.25 [3.42 – 11.52] | 1.11 [0.99 – 1.14] | →∞ |
|  | Compound | 0.24 | 0.12 [0.05 – 0.24] | 8.35 [4.79 – 14.08] | 1.21 [1.07-1.36] | 1.35 |
|  | Household | 0.25 | 0.29 [0.16 – 0.51] | 7.09 [3.58 – 13.73] | 1.22 [0.99 – 1.45] | 1.25 |
|  | Room | 0.10 | 1.34 [0.97 – 1.81] | 2.68 [1.24 – 5.51] | 0.93 [0.57 – 1.45] | 1.07 |
|  | | | | | | |
| Jali village, Kiang West district,  The Gambia | Compound | 0.73 | 0.76 [0.39 - 1.40] | 4.01 [1.81 – 7.38] | 1.05 [0.84 – 1.23] | 2.81 |
|  | Room | 0.30 | 1.66 [1.18 – 2.27] | 1.74 [0.56 – 3.91] | 0.63 [0.12 – 1.04] | 1.76 |
|  | | | | | | |
| Kahe Mpya sub-village, Rombo district,  Tanzania | Balozi | 0.90 | 0.70 [0.24 – 1.63] | 2.63 [0.09 – 87.4] | 0.99 [0.24 – 1.87] | 1.54 |
|  | Kaya  Room | 0.30  0.18 | 1.73 [1.18 – 2.37]  2.24 [1.70 – 2.88] | 1.57 [0.29 – 5.31]  0.87 [0.25 – 2.48] | 0.89 [0.06 – 1.63]  0.52 [0.00 – 1.42] | 1.18  1.18 |
|  | | | | | | |
| Maindi village, Kongwa district,  Tanzania | Balozi | 1 | 0.11 [0.003 – 1.25] | 8.43 [3.44 – 18.8] | 1.14 [0.95 – 1.34] | 805 |
|  | Kaya | 0.60 | 1.70 [1.15 – 2.46] | 3.06 [1.14 – 6.18] | 0.88 [0.41 – 1.26] | 2.65 |

Text S1 TABLE 1 Sensitivity of the transmission parameters estimates by definition of ‘household’ unit

| Community | Global transmission coefficient, *β_G_* [95% CI] | Local transmission coefficient,  *β_L_* [95% CI] | Coefficient for density dependence,  *w* [95% CI] | *R** |
| --- | --- | --- | --- | --- |
| 14 villages, Upper Saloum District, The Gambia | 0.36 [0.21 – 0.59] | 5.90 [2.86 – 11.91] | 1.17 [0.91 – 1.42] | 1.21 |
| Jali village, Kiang West District, The Gambia | 0.89 [0.48 – 1.56] | 3.76 [1.63 – 7.15] | 1.04 [0.82 – 1.24] | 2.53 |
| Sub-Village of Kahe Mpya, Rombo District, Tanzania | 1.73 [1.23 – 2.36] | 1.64 [0.32 – 5.51] | 0.91 [0.09 – 1.65] | 1.21 |
| Maindi village, Kongwa district, Tanzania | 1.64 [1.21 – 2.19] | 2.87 [1.25 – 5.40] | 0.92 [0.49 – 1.28] | 1.84 |

Text S1 TABLE 2 Estimation of transmission parameters, excluding individuals from the communities that were not examined

| Community | Probability ratio of uninfected : infected being sampled | Global transmission coefficient,  *β_G_* [95% CI] | Local transmission coefficient,  *β_L_* [95% CI] | Coefficient for density dependence,  *w* [95% CI] | *R** |
| --- | --- | --- | --- | --- | --- |
| 14 villages, Upper Saloum District, The Gambia | 0.5  1  2 | 0.29 [0.15 – 0.50]  0.29 [0.16 – 0.51]  0.30 [0.17 – 0.51] | 7.39 [3.72 – 14.20]  7.09 [3.58 – 13.73]  6.80 [3.49 – 13.37] | 1.23 [1.01 – 1.46]  1.22 [0.99 – 1.45]  1.21 [0.99 – 1.44] | 1.27  1.25  1.23 |
| Jali village, Kiang West District, The Gambia | 0.5  1  2 | 0.76 [0.38 – 1.41]  0.76 [0.39 – 1.40]  0.78 [0.41 – 1.41] | 4.06 [1.84 – 7.42]  4.01 [1.81 – 7.38]  3.92 [1.78 – 7.34] | 1.05 [0.84 – 1.23]  1.05 [0.84 – 1.23]  1.05 [0.84 – 1.23] | 2.98  2.81  2.68 |
| Sub-Village of Kahe Mpya, Rombo District, Tanzania | 0.5  1  2 | 1.73 [1.22 – 2.39]  1.73 [1.18 – 2.37]  1.72 [1.18 – 2.36] | 1.54 [0.28 – 5.28]  1.57 [0.29 – 5.31]  1.59 [0.30 – 5.38] | 0.88 [0.05 – 1.62]  0.89 [0.06 – 1.63]  0.90 [0.07 – 1.64] | 1.19  1.18  1.18 |
| Maindi village, Kongwa district, Tanzania | 0.5  1  2 | 1.74 [1.16 – 2.59]  1.70 [1.15 – 2.46]  1.63 [1.12 – 2.36] | 3.06 [1.13 – 6.15]  3.06 [1.14 – 6.18]  3.13 [1.20 – 6.28] | 0.87 [0.39 – 1.25]  0.88 [0.41 – 1.26]  0.90 [0.44 – 1.29] | 2.86  2.65  2.49 |

Text S1 TABLE 3 Estimation of transmission parameters, accounting for if infected individuals were half, equal or twice as likely to be sampled than uninfected individuals

**Text S1 FIGURE 2** Sensitivity of transmission parameters estimates by varying the duration of infection

1. Elliott JM (1977) *Some Methods for the Statistical Analysis of Samples of Benthic Invertebrates*. Cumbria: Freshwater Biological Assn
